# Intrathecal baclofen treatment in dystonic cerebral palsy: a randomized clinical trial: the IDYS trial

**DOI:** 10.1186/1471-2431-13-175

**Published:** 2013-10-28

**Authors:** Laura A Bonouvrié, Jules G Becher, Johannes SH Vles, Karin Boeschoten, Dan Soudant, Vincent de Groot, Willem JR van Ouwerkerk, Rob LM Strijers, Elisabeth Foncke, Joke Geytenbeek, Peter M van de Ven, Onno Teernstra, R Jeroen Vermeulen

**Affiliations:** 1 Department of Rehabilitation Medicine, VU University Medical Center, Postbus 7057, 1007, MB Amsterdam, The Netherlands; 2 Department of Child Neurology, Maastricht University Medical Center, Amsterdam, The Netherlands; 3 Department of Neurosurgery, VU University Medical Center, Amsterdam, The Netherlands; 4 Department of Clinical Neurophysiology, VU University Medical Center, Amsterdam, The Netherlands; 5 Department of Neurology, VU University Medical Center, Amsterdam, The Netherlands; 6 Department of Epidemiology and Biostatistics, VU University Medical Center, Amsterdam, The Netherlands; 7 Department of Neurosurgery, Maastricht University Medical Center, Amsterdam, The Netherlands; 8 Department of Child Neurology, Neuroscience Campus Amsterdam, VU University Medical Center, Amsterdam, The Netherlands

**Keywords:** Cerebral palsy, Dystonia, Dyskinesia, Goal attainment scaling, Intrathecal baclofen, Randomized controlled trial

## Abstract

**Background:**

Dystonic cerebral palsy is primarily caused by damage to the basal ganglia and central cortex. The daily care of these patients can be difficult due to dystonic movements. Intrathecal baclofen treatment is a potential treatment option for dystonia and has become common practice. Despite this widespread adoption, high quality evidence on the effects of intrathecal baclofen treatment on daily activities is lacking and prospective data are needed to judge the usefulness and indications for dystonic cerebral palsy. The primary aim of this study is to provide level one clinical evidence for the effects of intrathecal baclofen treatment on the level of activities and participation in dystonic cerebral palsy patients. Furthermore, we hope to identify clinical characteristics that will predict a beneficial effect of intrathecal baclofen in an individual patient.

**Methods/Design:**

A double blind placebo-controlled multi-center randomized clinical trial will be performed in 30 children with dystonic cerebral palsy. Patients aged between 4 and 25 years old with a confirmed diagnosis of dystonic cerebral palsy, Gross Motor Functioning Classification System level IV or V, with lesions in the cerebral white matter, basal ganglia or central cortex and who are eligible for intrathecal baclofen treatment will be included. Group A will receive three months of continuous intrathecal baclofen treatment and group B will receive three months of placebo treatment, both via an implanted pump. After this three month period, all patients will receive intrathecal baclofen treatment, with a follow-up after nine months. The primary outcome measurement will be the effect on activities of and participation in daily life measured by Goal Attainment Scaling. Secondary outcome measurements on the level of body functions include dystonia, spasticity, pain, comfort and sleep-related breathing disorders. Side effects will be monitored and we will study whether patient characteristics influence outcome.

**Discussion:**

The results of this study will provide data for evidence-based use of intrathecal baclofen in dystonic cerebral palsy.

**Trial registration:**

Nederlands Trial Register, NTR3642

## Background

### Dystonic cerebral palsy

Cerebral palsy (CP) is a group of disorders caused by non-progressive disturbances that occurred in the developing fetal or early infant brain. The classification of cerebral palsy includes the classical neurological terms for central motor disorders: spasticity, dyskinesia and ataxia [1,2]. Dyskinesia can be further differentiated into dystonia and choreoathetosis [2]. Dystonia is described as an abnormal pattern of posture and/or movement that is involuntary, uncontrolled, recurring and occasionally stereotyped. These movements can interfere with daily care and may be painful and uncomfortable. The dyskinetic form of cerebral palsy, including the dystonic form, is in most patients caused by lesions in the basal ganglia. Additional lesions of the central cortex are found in some cases. This type of brain damage is a common pattern in asphyxiated infants born at term [3].

### Treatment

The results of pharmacological treatment of severe dystonic CP have been rather disappointing. Positive effects on dystonia with levodopa, anticholinergic drugs or muscle relaxants including benzodiazepines and baclofen, have been reported by some authors [4,5]. In addition, Albright and co-workers described an anti-dystonic effect of intrathecal baclofen (ITB) treatment. Despite the fact that studies on the effects of ITB on dystonic cerebral palsy are limited in number and the level of evidence is low, ITB is now common practice in the treatment of severe dystonic CP.

Not all patients are eligible for ITB treatment. The following criteria apply: 1. The etiology is preferably known; 2. Management of aggravating factors, such as pain and discomfort, should be optimal; 3. Other treatment options should have been explored. Oral pharmacological treatment with levodopa, anticholinergic drugs or muscle relaxants including benzodiazepines and baclofen must have been attempted and must have resulted in high oral dosages with either unacceptable side effects or insufficient efficacy; 4. The movement disorder should be so severe that it interferes with activities of daily life or quality of life; 5. Treatment goals should be clear and applicable and, to avoid disappointment, it is important that patients and parents understand these goals; 6. Patients and parents should be motivated and able to adhere to the requirements of treatment, such as the frequent pump fillings and checkups in the outpatient clinic; 7. Patients should have sufficient body size to allow pump implantation [6].

### Objectives

The primary objective of the present study is to show whether ITB treatment improves activities of and participation in daily life (for example dressing, transfer, sitting in a wheelchair, hygienic care, speech) in dystonic CP patients. Certain patient characteristics, such as the location and severity of MRI lesions and Gross Motor Functioning Classification Score (GMFCS) level, might influence the effects of ITB treatment in patients with dystonic CP. Therefore, we will study whether individual patient characteristics (GMFCS, gender, age, MRI findings and co-medication) influence outcome and could be used in determining the indication for ITB in future patients. A secondary objective is to provide evidence for the effect of ITB on the level of body functions. The relevant clinical questions to be addressed are: 1. Does ITB decrease dystonia? 2. Does ITB decrease spasticity in dystonic patients? 3. Does ITB decrease pain? 4. Does ITB increase comfort? 5. Does ITB influence screening results of sleep-related breathing disorders? 6. What are the side effects of ITB?

## Methods/Design

### Study design

**Figure 1:**
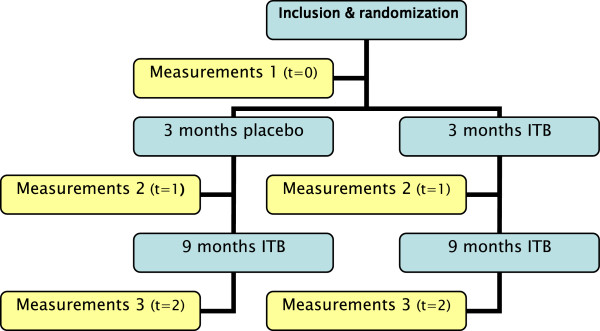
Flow chart subjects.

The design of the study is a double blind placebo-controlled multi-center randomized clinical trial. It will be conducted in the VU University Medical Center (VUMC) in Amsterdam and the Maastricht University Medical Center (MUMC) in Maastricht (both in the Netherlands). The Medical Ethical Committee of the VU University Medical Center approved the study. In both centers local practicability was granted subsequently. Subjects will be included over a period of two and a half years and they will participate in the study for one year. Figure 1 shows the flow scheme for subjects and timing of measurements throughout the study.

### Participants

Thirty subjects will be recruited from the outpatient clinics of the pediatric neurology and pediatric rehabilitation departments of the VUMC and the MUMC. Table 1 shows the inclusion and exclusion criteria. We selected ages between 4 and 25 years old because older patients often show secondary complications, such as contractures, that could introduce greater variation into both the effects of treatment and treatment goals. Furthermore, to achieve a homogeneous patient group, only GMFCS IV and V (non-walkers) will be included. All patients and/or their caregivers will sign an informed consent form before participating in the study.

**Table 1:** Summary of inclusion and exclusion criteria

**Inclusion criteria**	**Exclusion criteria**
• Dystonic cerebral palsy	• Contra-indications for general anesthesia
• GMFCS IV or V	• Contra-indications for baclofen
• Eligible for ITB treatment using criteria of common practice	• Oral pharmacological treatment is sufficient
• Lesions on MRI (cerebral white matter, basal ganglia, central cortex)	• Inadequate knowledge of Dutch language
• Aged 4 to 25 years old	• Deep brain stimulation
• Able and willing to complete study protocol	• Ventriculoperitoneal drain
• Consensus about inclusion	• Other disorders interfering with treatment

### Sample size calculation

We will use goal attainment scaling (GAS) as our primary outcome measure. The within group change for the placebo group is anticipated to be 0, with a standard deviation (SD) of 8.5. For the intrathecal baclofen group, the within group change is anticipated to be 12.5, with a SD of 10. To achieve a power of 90% when testing for a difference in means using a two-tailed independent samples t-test and a significance level of 5%, a total of 13 subjects per group will be needed.

In a previous study in the VU University Medical Center, 4 infections occurred in 34 pump implantations [7]. Ward and coworkers reported pump removal in 5.9% of the cases with infections following implantation [8]. Extrapolating these numbers, inclusion of 30 patients is expected to lead to 3.5 infections and 1.2 pump removals. To prevent the study from becoming underpowered, due to unexpected protocol violations or subject dropout due to complications other than those related to intrathecal baclofen treatment, we will include 15 subjects per group.

### Intervention

Included subjects will be randomized in two groups. Group 1 will receive placebo treatment via an implanted micro-infusion pump for three months. Group 2 will receive ITB treatment via an implanted micro-infusion pump for three months. In our opinion, it is unethical to continue placebo treatment for more than three months. If interim analysis shows that subjects in one of the groups have significant disadvantages compared to the other group, the study will be prematurely terminated. On the other hand, we know from experience that dosages for some patients are still being modified three months after starting treatment. As a consequence, the effect of treatment in these patients may not yet be optimal, which may then result in no detectable effect of ITB treatment at that point. We will try to reduce this possible effect through frequent dose modification. Patients will be seen once or twice weekly to increase their dosage, until either a stable dosage or a maximal dosage of 800 μg/day is achieved.

Subjects will be assessed at three months and the effect of treatment in the two groups compared. Since determining the most beneficial pump setting can take more than three months and because the initial effect may recede in some cases, all subjects will receive subsequent ITB treatment for nine months. Following this 9 month period, clinical evaluation will be performed in order to determine long-term effects (see Figure 1. Flow Chart of Subjects).

### Dosage

Patients with dystonia seem to require larger ITB doses than the doses used to treat spasticity [9]. Albright and colleagues noticed a change in dystonia only after 4 days of continuous infusion [10]. The effective dose of ITB was in the range of 350 to 750 μg/day [9-11]. Since a screening period will not be included, we do not know how subjects will respond and which dose will be sufficiently effective. Therefore, the starting dosage will be 50 μg/24 h.

Dosage can be increased by 10-20% daily. When subjects are discharged from the hospital following pump implantation, they will be seen once a week or once every two weeks for dosage modification. After three weeks of increasing dosage, with disappearance of the effect of the increase, daily boluses will be administered to a maximum of four times a day, with a minimal interval of four hours. For the blinded part of the study, a maximum dosage of 800 μg/day will be administered. This is a reasonable estimation of the maximal dosage after three months. Since treatment during the study is blinded, the physician in charge of regular follow-up after pump implantation, including pump filling and dosage adjustments, will be unaware of the subject’s allocated group. To provide optimal treatment, pump settings will be individually altered to the extent that the physician would consider necessary, based on the findings of physical examination and parental and patient interview, as were the patient not participating in the study. As a consequence, the pump settings of subjects in the placebo group will be altered as if the patient was receiving ITB treatment.

Lack of response to ITB can be caused by pump or catheter dysfunction. Placebo treatment may also cause lack of effect and for this reason no action will be taken during the first three months of the study in cases where pump or catheter dysfunction would normally be suspected. An exception will be made when the condition of the patient requires otherwise, such as in the case of signs of withdrawal. One week after pump implantation, all patients will undergo X-ray of the spine to determine the position of the catheter tip. The catheter should be placed approximately at the fourth cervical level, and at least above the level of Th1.

### Outcome measures

Outcome measurements are defined on the levels of the International Classification of Functioning, Disability and Health (ICF) model of the World Health Organization (WHO). We distinguish the level of activities and participation and the level of body functions. Body functions are the physiological and psychological functions of body systems. Activities are defined as the execution of a task or action by an individual. Participation is involvement in a life situation [12].

### Primary outcome measure

The primary outcome measurement will be on the level of activities and participation. Goal attainment scaling (GAS) will be used to measure the effect of ITB treatment. Using GAS, achievement of individual set goals can be quantified. This method was introduced for assessing outcomes in mental health settings and has been used in many other areas [13,14]. Each subject has their own outcome measure, but statistical analysis is possible because they are scored in a standardized way. This procedure is time-consuming and requires about 45 minutes per child [15,16]. However, after the scale is constructed, it should be possible to score a patient within 10 minutes [17].

The procedure includes the following aspects:

1) Identification of goals:

Three main problems of daily care and function will be determined by caregivers. Goals will be set for these problems with the help of the team to ensure that goals are achievable. Goals should be specific, measurable, attainable, realistic and timely (SMART) [13,14]. The target activity, specific support and time period should be specified and performance should be quantified using distance, frequency or time taken to accomplish a task [14].

2) Weighing of goals:

Some goals will be more important for subjects than others [13]. Goals can be assigned a weighing score by caregivers and a difficulty score by the team [14]. We chose not to assign weighing scores since the weighted and unweighted scores are closely correlated [18]. A value of 1 is applied to ‘weight’ in the formula described below.

3) Definition of expected outcomes:

Several approaches are described in literature, varying from 5 to 7 point scales [14,15]. We will use a 6 point scale ranging from -3 to +2, since we wish to include both the possibility of partially achieving the set goal and to avoid a bottoming effect. Baseline scores will be allocated as -2. If the subject achieves the expected level, this is scored as 0. If a subject does not achieve the expected level but shows improvement, this is scored as -1. If they achieve more than the expected outcome, this is scored as +1 for somewhat more and +2 for much more. We chose to add a score of -3 in case of deterioration. Each goal level will be defined by the investigator so as to be as objective and observable as possible [13].

4) Scoring goal attainment:

For each assessment, one assessor will make a standardized video recording during trials for each of the three functional ability goals. The recording procedure will be identical for all measurements. Another assessor, blinded for group allocation, will rate the subject’s performance from the video recordings. Although the GAS only simulates the subject’s own functional setting, parents were convinced that the outcome of scaling was representative of their own setting [19].

Goal attainment scores will be recorded at baseline, after three months of blinded treatment and after nine months of ITB treatment. Goal attainment change scores will be determined by subtracting the baseline score from the outcome score. Subjects who achieve a GAS T-score >50 achieved their goals [8]. Clinical relevance will be defined as an improvement of at least two points in at least one of the three goals [19].

A single aggregated score (T-score) can be produced by a standardized mathematical formula: Overall GAS = 50+ 10∑(w_i_x_i_) / √((1-ρ) ∑w_i_^2^ + ρ(∑w_i_)^2^); W_i_ is the weight assigned to the goal, x_i_ is the numerical value achieved (between -3 and +2), ρ (rho) is the expected correlation of the goal scales, which is normally 0.3 [13,14].

### Secondary outcome measures

#### Dystonia

Videos of all patients will be made using the video protocol described by Monbaliu et al. [20]. Blinded therapists or physicians will assess all videotapes and rate dystonia using the Barry-Albright dystonia (BAD) scale and the Dyskinesia Impairment Scale (DIS).

The BAD scale is a five point ordinal severity scale to assess secondary dystonia in eight body regions (eyes, mouth, neck, trunk, each upper and lower extremity) [21]. Raters score dystonia as none, slight, mild, moderate or severe. A reduction of 25% or more, in comparison with the baseline score, is considered clinically significant. In our experience, interrater variability is high, but BAD scores are generally accepted as a measurement of dystonia and are widely used to assess dystonia.

Recently, a new instrument to measure dystonia in dyskinetic CP became available, the DIS [20]. It consists of two subscales: dystonia and choreoathetosis. Scoring is carried out in 12 body regions all in two conditions (rest and activity). Both duration and amplitude are evaluated. Since this is a new instrument, we will use the DIS in addition to the BAD.

#### Electromyography

The DIS has no external validation. We decided that Surface Electromyography (EMG) might provide an impression of muscle activity level underlying dystonia. Therefore, surface EMG measurements will be carried out to determine mean EMG activity in individually determined muscle groups and in multiple conditions such as rest and during activities.

#### Spasticity

The soleus Hoffmann-reflex (H-reflex) represents excitability of the neural components of the stretch reflex arc [22]. The H/M-ratio of the H-reflex represents an increased excitability of soleus motor neurons. The H/M-ratio is increased in subjects with spasticity due to various origins, [23] and it has been shown that the H_max_ decreases significantly after ITB administration in children with spastic cerebral palsy when compared with baseline measurements. This represents a decrease in motor neuron excitability. Furthermore, the response appears to be dose-dependent [22]. Although not all children tolerate the measurements, feasibility of the H-reflex was 93% in a study by Hoving and co-workers [22].

For clinical assessment of spasticity in children with central motor disorders, the spasticity test (SPAT) is used during standard physical examination. When using the SPAT, we will follow a standardized protocol as described in the guideline for standard physical examination of children with central motor disorders [24,25]. The difference between the range of motion and the angle of catch will be used as the outcome measure for spasticity [26]. The test takes approximately 5 to 8 minutes per limb to perform [25]. This test might be difficult to perform in children and adolescents with severe dystonia and its usefulness will become evident during the study.

#### Pain and comfort

Parents will score pain and comfort on a visual analogue scale (VAS). The VAS is a straight 10 cm long horizontal block consisting of 10 smaller blocks with anchor points at 0 and 10. For pain, a score of 0 represents ‘no pain’ and a score of 10 represents ‘the worst possible pain’. For comfort, a score of 0 will represent being ‘very uncomfortable’ and a score of 10 is having ‘no problems at all’. If patients are able to indicate their mood, it will be scored by pointing out the applicable happy face, with choices of six faces ranging from very happy to very sad.

#### Sleep-related breathing disorders

Children with CP have a higher risk of sleep-related disorders than typically developing children [27]. Bensmail et al. showed that patients with severe spasticity due to spinal cord injury and multiple sclerosis, treated with ITB by bolus administration, showed an increased respiratory disturbance index (the number of apneas/hypopneas per hour of sleep) [28,29]. Polysomnography is the gold standard when measuring sleep-related breathing disorders (SRBD). However, this time-consuming and burdensome diagnostic test is not practical for children participating in research [30]. A subscale of the Pediatric Sleep Questionnaire was developed to measure SRBD. This scale consists of 22 items and can be completed in five minutes. Sensitivity and specificity are high (81% and 87%). The scale is positive for a high risk of SRBD when there are 8 or more positive answers to the 22 question items (≥33%) [30]. As the burden of a questionnaire is low for the patients and their families, as compared to polysomnography, we will use this questionnaire to determine if ITB changes the risk of SRBD.

#### Classification

For classification of the severity of motor abilities we will use the GMFCS [31,32] and the Manual Ability Classification System (MACS) [33,34]. GMFCS and MACS classification will be scored at baseline, 3 months and at twelve months. In a study by Voorman and co-workers, 74% of children with CP had restrictions in communication [35]. Since many children with severe CP (GMFCS IV and V) cannot speak, assessment of language abilities cannot be based on speech production. In addition, language comprehension skills are difficult to assess in children with severe CP. Therefore, we will use the Computer-Based Instrument for Low motor Language Testing (C-BiLLT) to measure comprehension of spoken language at baseline. The validity of this instrument has been tested [36]. We do not expect changes in outcomes on the C-BiLLT with ITB treatment since comprehension of spoken language is highly correlated with cognition, and cognition is not effected by ITB treatment. We will use the outcome of the C-BiLLT as a patient characteristic.

Magnetic resonance imaging (MRI) will be used to classify the severity of damage to the gray matter structures (cortex and basal ganglia) and white matter (loss of white matter and gliosis). The severity of brain damage on MRI can be classified in three groups; mild, moderate and severe. The mild pattern includes involvement of nucleus lentiformis and ventro-lateral thalamus, the moderate pattern includes additional involvement of the peri-central region and the severe pattern includes additional involvement of the entire thalamus and hippocampus. In the mild and moderate damage groups, infants suffer from the dyskinetic form of cerebral palsy, whereas the severe type of damage frequently produces purely spastic paresis [3]. MRI studies of the brain are necessary to confirm the diagnosis of cerebral palsy. If MRI studies have been conducted previously, these studies will be critically assessed. If the quality is good, the MRI will be accepted and no further imaging is needed. If the quality of the MRI images is poor, the patient will undergo a new MRI including diffusion tensor imaging (DTI). If a patient had not yet undergone a MRI, one will be made including DTI. Adding DTI images to a regular MRI scan will require an extra scanning time of approximately four minutes. In this patient category MRI imaging has to be done under general anesthesia, since dystonic movements interfere with MRI quality.

#### Other study parameters

To assess the safety of ITB treatment, side effects and complications will be closely monitored. The complication rate will be calculated by dividing the number of complications by the duration in years of pump implantation.

Functional abilities at baseline will be assessed by the Paediatric Evaluation of Disability Inventory (PEDI) and will be repeated after three and twelve months. The PEDI was developed for children from six months to seven and a half years of age and assesses skills in mobility, self-care and social function. It can also be used for older children if their functional abilities are expected to be below the level of a child of seven and a half years old. The functional scales indicate if children with disability are able or unable to perform certain activities. Separate measures assess the degree of caregiving assistance and equipment modification that is needed to accomplish complex functional skills. Scores on the caregiver assistance scale are noted on a range from independent to maximal assistance and modification scores are scored as none, child-oriented, rehabilitation-oriented or extensive [37,38]. The PEDI will be administered through direct assessment by a therapist.

### Randomization, blinding and treatment allocation

Subjects will be randomized in two groups by block randomization. Randomization will be done by the pharmacies of the VUMC and the MUMC. The pharmacist will be the only holder of the code for randomization. In case of emergency, the code will be accessible 24 hours per day and 7 days per week via either the pharmacist of the VUMC or MUMC or by opening a sealed envelope containing the subject’s group, available at the departments concerned. Allocation will be concealed, and the researchers, assessors and the physician responsible for pump filling will be blinded.

### Premature termination

#### Withdrawal of individual subjects

Subjects can end participation in the study at any time without providing a reason and without consequences for their future treatment in the clinic. The investigator can decide to withdraw a subject from the study for urgent medical reasons. Subjects withdrawn from the study will continue their regular follow-up outside of the study protocol. Subjects will be replaced if they withdraw before pump implantation has taken place. Subjects who withdraw after pump implantation has taken place will not be replaced. With subject agreement, a final assessment will take place before definite ending of participation. These subjects will not be included in the analysis but we will present a fact sheet including their information.

#### Data safety monitoring board

A data safety monitoring board will be formed and will meet periodically to review aggregate and individual subject data related to safety, data integrity and overall conduct of the trial. They will assess the risk/benefit balance, including a statistical analysis if necessary. Depending upon this assessment, the board will provide recommendations to continue, adapt or terminate the trial.

### Statistics

#### Descriptive statistics

Patient characteristics will be described. Gender distribution will be compared between the ITB and placebo group using a Chi-square test. Mean age between the ITB and placebo group will be compared using an independent samples t-test. Means and standard deviation (SD) of the PEDI scores, C-BiLLT scores, GAS t-score, BAD score, SPAT score and mean EMG activity at baseline will be tabulated. Means and SD of the GAS t-score, BAD score, DIS score, SPAT score and mean EMG activity at follow up will be separately tabulated per group.

#### Univariate analysis

The GAS t-scores, BAD score, SPAT score and EMG activity in the placebo and ITB group will be compared using an independent samples t-test. If the assumption of normality appears not to be valid, the non-parametric Mann–Whitney test will instead be used. A p-value of 0.05 is considered statistically significant for this primary analysis. To assess within-group changes in means between follow-up times and baseline GAS t-scores, BAD score, DIS score, SPAT score and EMG activity, a paired t-test or non-parametric Wilcoxon test will be used (depending on whether the normality assumption is valid). In these analyses, a p-value of 0.05 is considered to be statistically significant.

#### Multivariate analysis

A multivariate analysis will be used to determine the effect of ITB treatment (primary: functional outcome; secondary: dystonia and the interaction with GMFCS, MACS, MRI classification and use of co-medication).

## Discussion

We anticipate that the results of this study will allow evidence-based use of intrathecal baclofen in dystonic cerebral palsy.

## Abbreviations

CP: Cerebral palsy; GMFCS: Gross motor functioning classification system; VUMC: VU University medical center; MACS: Manual ability classification System; EMG: Electromyography; MUMC: Maastricht University medical center; ITB: Intrathecal baclofen; MRI: Magnetic resonance imaging; SD: Standard deviation; PEDI: Pediatric evaluation of disability inventory; RM: Repetitive movements; GAS: Goal attainment scaling; BAD: Barry albright dystonia scale; DIS: Dyskinesia impairment scale; C-BiLLT: Computer based instrument for low motor language testing; dti: Diffusion tensor imaging; VAS: Visual analogue scale; ICF: International classification of functioning, disability and health.

## Competing interests

There are no competing interests or financial competing interests.

## Authors’ contributions

LB participated in the design and drafted the manuscript. JB and RV participated in the design and first drafts of the manuscript. JV, KB, DS, VG, WO, RS, EF, JG, PV, OT participated in reviewing the design. All authors participated in the reviewing process and approved the final manuscript.

## Pre-publication history

The pre-publication history for this paper can be accessed here:

http://www.biomedcentral.com/1471-2431/13/175/prepub
